# A modification of the leaf-bags method to assess spring ecosystem functioning: benthic invertebrates and leaf-litter breakdown in Vera Spring (Central Italy)

**DOI:** 10.7717/peerj.6250

**Published:** 2019-02-13

**Authors:** Giovanni Cristiano, Bruno Cicolani, Francesco Paolo Miccoli, Antonio Di Sabatino

**Affiliations:** 1 Department of Life, Health and Environmental Sciences, University of L'Aquila, L'Aquila, Italy; 2 Department of Civil, Construction-Architecture & Environmental Engineering, University of L'Aquila, L’Aquila, Italy

**Keywords:** Poplar leaves, Detritus processing, Macroinvertebrates, Functional attributes, Spring ecosystems, Biocoenotic interactions

## Abstract

The evaluation of leaf detritus processing (decomposition and breakdown) is one of the most simple and cost-effective method to assess the functional characteristics of freshwater ecosystems. However, in comparison with other freshwater habitats, information on leaf litter breakdown in spring ecosystems is still scarce and fragmentary. In this paper, we present results of the first application of a variant of the leaf-bags method to assess structure of macroinvertebrate assemblages and leaf-litter breakdown in a Central Apennines (Italy) cold spring which was investigated from July 2016 to October 2016. Notwithstanding the stable conditions of almost all hydrological and physico-chemical parameters, we found significant temporal differences in (i) % of mass loss of poplar leaves (ii) number of Ephemeroptera, Plecoptera and Trichoptera taxa, (iii) shredder and predator densities. We demonstrate that detritus processing in cold springs may be faster than or as fast as in warmer streams/rivers. Shredders activity and biocoenotic interactions, rather than temperature and nutrients load, were the main drivers of the process. A routine application of the modified leaf-bags may contribute to expand our knowledge on detritus processing in cold springs and may help to predict impacts of climate warming on freshwater ecosystem functioning.

## Introduction

Recent studies have highlighted the importance of structural and functional parameters to assess the ecological integrity of freshwater ecosystems (Young, Matthaei & Townsend, 2008; Woodward et al., 2012). The evaluation of leaf detritus processing (decomposition and breakdown) is one of the simplest and most cost-effective method to assess the functional characteristics of lotic and lentic systems and has been successfully employed in a wide range of aquatic habitats under natural and anthropogenic disturbance (Chauvet et al., 2016; Di Sabatino et al., 2014; Gessner & Chauvet, 2002; Pinna et al., 2016). Data on the relative importance of biotic and abiotic factors in affecting plant litter decomposition are also essential to predict impacts of climate warming on freshwater ecosystem functioning and global carbon fluxes (Boyero et al., 2011, 2014, 2016; Jyväsjärvi et al., 2015; Follstad Shah et al., 2017). However, compared to other freshwater habitats, data on leaf-litter breakdown in spring/springbrook habitats are still scarce and fragmentary (Bartodziej & Perry, 1990; Robinson & Gessner, 2000; Sangiorgio et al., 2010; Benstead & Huryn, 2011; Lehosmaa et al., 2017), mostly because of the lack of standardized methodologies.

Most of the available methods for spring ecological assessment are mainly focused to evaluate biodiversity or spatial and temporal variation of crenic assemblages, rarely allowing for a concomitant evaluation of functional parameters (Di Sabatino et al., 2018). Therefore, excluding some older seminal papers (Odum, 1957; Teal, 1957; Tilly, 1968; Williams & Hogg, 1988) information on ecosystem-level processes in springs is still rudimentary (Robinson et al., 2008).

Recently, Di Sabatino et al. (2018) proposed the leaf-nets as a new quantitative method for sampling benthic invertebrates in spring habitats and demonstrated that, compared to other active and passive methods, leaf-nets were effective in describing the organization of spring assemblages with negligible impacts on microhabitats and populations of such delicate and fragile ecosystems. The leaf-nets are essentially a slight modification of the widely used leaf-bags and may be employed for a concomitant evaluation of structural and functional attributes (detritus processing) of spring ecosystems. The present paper thus aims (i) to demonstrate how the modified leaf-bags can be successfully used to assess leaf-litter breakdown in springs, (ii) to add new data on the rate of leaf detritus processing in spring habitats, and (iii) to make some preliminary considerations on the main factors affecting detritus breakdown in cold springs. We predicted that detritus decomposition in hydrologically and physico-chemically stable springs would be mainly driven by biotic factors and that the modified leaf-bags would allow a reliable ecological characterization of these ecosystems.

## Materials and Methods

### Study site

The study area was located at “Capo Tempera” (coordinates 42°22′21.42″N, 13°27′30.51″E; altitude 664 m asl), one of the main resurgences of the complex of karst–limestone “Vera Springs” (L’Aquila, Abruzzo, Central Italy). The spring (rheocrene) is mostly shaded by dense riparian vegetation (*Populus nigra* and *Salix alba)* and is characterized by low concentration of nutrients, stable discharge (∼0.3 m^3^ s^−1^), almost constant temperature (∼8 °C) and slight variation in other physico-chemical parameters (Cristiano, 2015; Di Sabatino et al., 2018). For a more detailed description of the study area see Cristiano (2015). Field experiments were authorized by the Managing Committee of Vera Spring natural reserve.

### Construction technique, field, and laboratory procedures

**Figure 1:**
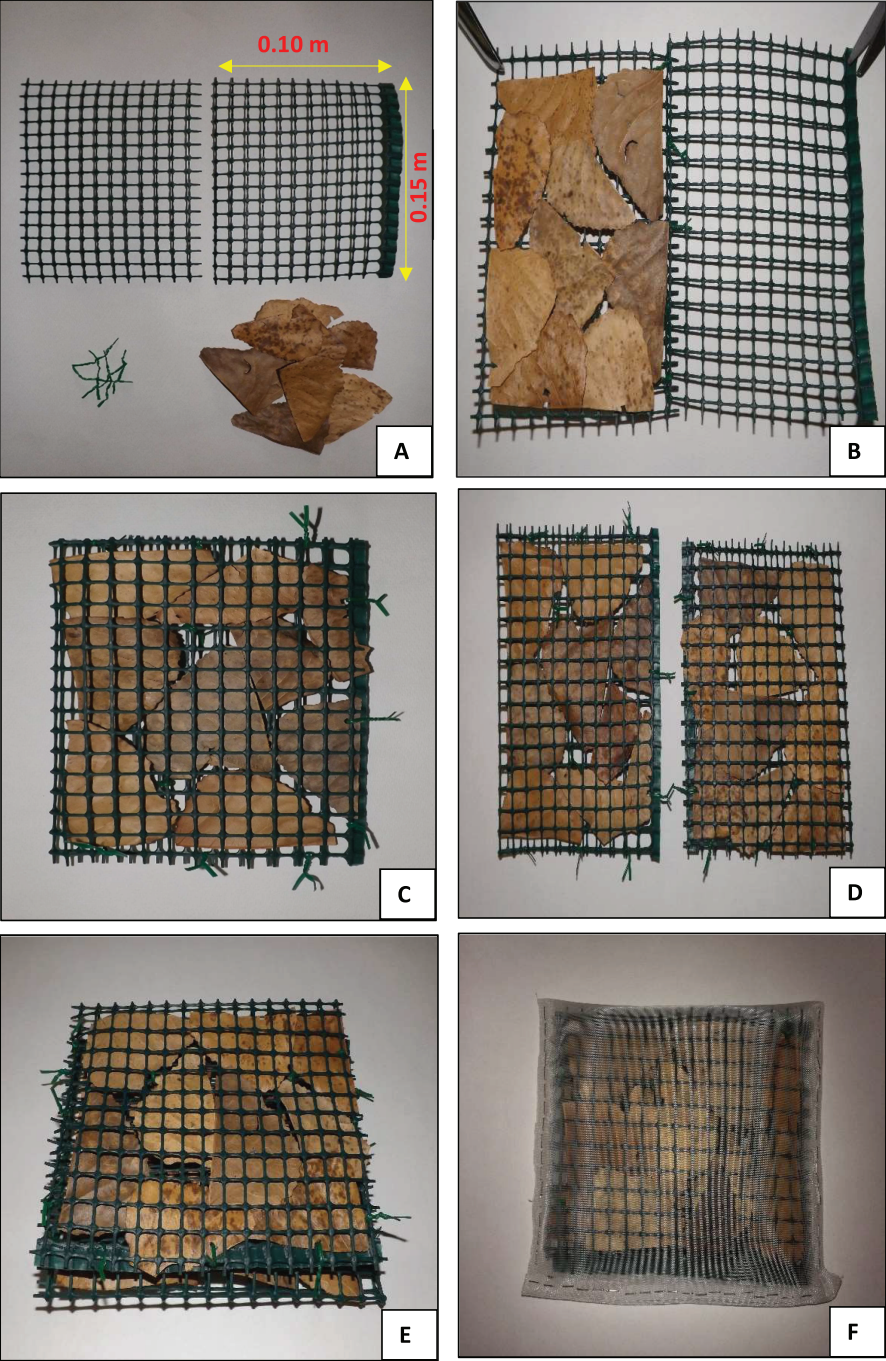
Leaf-nets. Photos showing the construction details of the modified leaf-bags; (A–E) coarse mesh (0.01 m), (F) fine mesh (500 μm)-not utilized in the present study. See text for more explanations.

Leaf-litter breakdown in Vera Spring was assessed by using the modified leaf-bags method (Di Sabatino et al., 2016, 2018). The device is formed by two PVC nets (0.10 m * 0.15 m, mesh size 0.01 m—Fig. 1A) filled with a layer of previously dried *P. nigra* leaves (Figs. 1B and 1C). Two of these units (area of 0.06 m^2^) were assembled using plastic coated steels (Figs. 1D and 1E); but see Di Sabatino et al. (2016, 2018) for details on construction technique and materials used.

In total, 24 bags were assembled and numbered for the subsequent identification after the retrieval phase. For each bag, the initial dry mass of poplar leaves (∼3 g) was determined with an analytical balance (±0.01 g).

In June 2016, six of these modified bags were randomly placed near the spring source (depth ∼0.2 m; current velocity ∼0.3 m s^−1^), firmly anchored to the spring bottom by using steel rods or fishing sinkers or boulders and stones, in relation to substrate coarseness. The retrieval phase, after 33 days of submersion, was conducted with care to avoid loss of organisms and residual leaf material. The leaf-bags were gently removed from their anchorage structures and immediately placed in a white tray. The residual material on the tray were put in plastic bags, labelled and transported to the laboratory in a portable cooler. This sampling procedure was repeated monthly from July 2016 to October 2016. In the laboratory, leaf-bags were gently washed, and macroinvertebrates were separated from the remaining leaf detritus. Poplar leaves were left for a week on a layer of absorbent paper in a dry and ventilated laboratory room and finally dried in a thermostatic oven at 60 °C for 72 h. The amount of remaining dry mass of poplar leaves was recorded with an analytical balance (±0.01 g) and these data were used to determine the dry mass loss of *P. nigra* detritus. The decomposition rate (*k*) was calculated according to Petersen & Cummins (1974), following the widely used exponential model of Olson (1963): *M_t_* = *M*_0_ * e^–*k t*^ where *M*_0_ is the initial dry mass, *M_t_* represents the remaining dry mass, and *t* is the time of immersion (33 days). We also calculated the half-life time of poplar detritus consumption as *t*_50_ = 0.693/*k*, the turnover time as *T* = 1/*k* and the percentage of daily mass loss as (1 − e^−*k*^) * 100 (Pinna et al., 2003).

All macroinvertebrate organisms sampled were stored in 70% ethanol and were counted and identified to the lowest taxonomic level (family, genus, or species) with a stereo microscope (Leica MZ9.5). Each taxon was assigned to a Functional Feeding Group (FFG) according to Merritt & Cummins (1996) and Tachet et al. (2000). At each sampling occasion, some physico-chemical parameters were recorded by using a multiparameter probe (Hach-Lange HQ40D multi). Concentrations of N−NO_3_^−^ and reactive-P were measured in laboratory following spectrophotometric standard procedures (Hach DR-2000).

### Statistical analysis

We assessed temporal differences in leaf detritus breakdown by applying one-way analysis of variance (ANOVA) with % of dry mass loss of poplar leaves as dependent variable and “month” (four levels: July, August, September, October) as discriminating factor. The data were analyzed in the context of a balanced design (*n* = 6 for all levels). ANOVA was also applied to detect differences in the structure of assemblages using taxa richness, number of Ephemeroptera, Plecoptera and Trichoptera (EPT) taxa and invertebrate density (log (*x*+1) transformed) as dependent variables. Assumptions of data normality and homogeneity of variances were previously verified with Anderson–Darling test and Levene test, respectively. Tukey’s post hoc test was applied when significant differences were detected by ANOVA.

The composition of macroinvertebrate assemblages in leaf-bags retrieved from July 2016 to October 2016 was compared using permutational multivariate analysis of variance (PERMANOVA) on the matrix of Bray–Curtis similarity, after log (*x*+1) transformation of taxa densities and the non-metric multidimensional scaling (nMDS) method was applied to plot the observed differences. PERMANOVA was also applied to detect differences in the functional organization of assemblages (Bray–Curtis similarity on log (*x*+1) transformed FFG densities). For each FFG, differences among the four periods were also assessed using one-way ANOVA after log (*x*+1) transformation of original densities.

Statistical analyses were performed with XLSTAT 2014.1.09 and PRIMER v6.1.16 & PERMANOVA + v1.0.6. The significance threshold for all analyses was set at *p* = 0.05.

## Results

Waters of Vera Spring are characterized by almost constant temperature, pH, conductivity, and Oxygen content and by low nutrient concentrations (Table 1).

**Table 1:** Physico-chemical parameters of Vera Spring.

Parameter	min	max	mean (±SD)
Temperature (°C)	8.0	8.5	8.3 (0.2)
pH	7.3	7.8	7.6 (0.2)
Conductivity (μS cm^−1^)	279	286	282 (3)
Dissolved oxygen (mg L^−1^)	10.3	10.5	10.4 (0.1)
N−N0_3_^−^ (mg L^−1^)	0.43	0.61	0.53 (0.08)
Reactive-P (mg L^−1^)	0.012	0.014	0.013 (0.001)

**Note:**

Mean values and range of variation of some physico-chemical parameters recorded in Vera Spring during the 4-months period of investigation.

**Figure 2:**
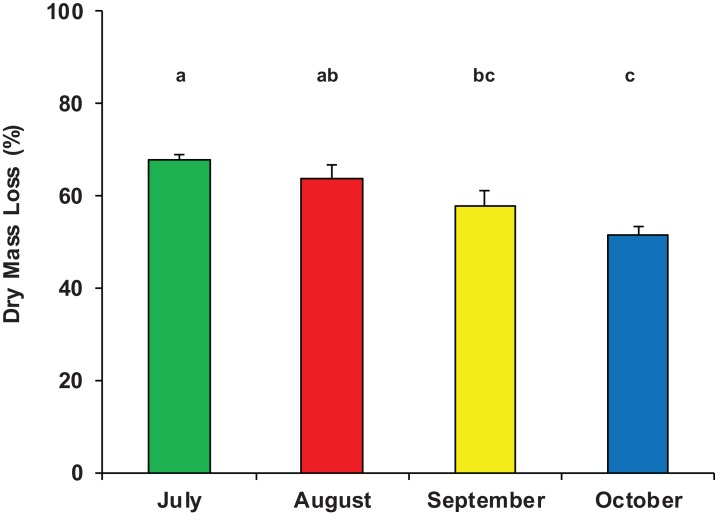
Dry mass loss of poplar leaves in Vera Spring. Mean (+1 SE) % dry mass loss of poplar leaves in modified leaf-bags retrieved from Vera Spring after 33 days of incubation, from July 2016 to October 2016. Letters above bars indicate significant differences after Tukey’s post hoc test.

On average, after 33 days of immersion, the mass loss of *P. nigra* leaves was 1.73 ± 0.40 g, equivalent to 60% ± 8% of the initial dry mass (Data S1) and accounting for a mean breakdown rate of *k* = 0.0285 ± 0.0063 day^−1^ (range: 0.0178–0.0383 day^−1^). The half-life (*t*_50_) of the initial leaf mass was on average 24 days, the percentage of daily mass loss was 3% and the turnover time (*T*) was 35 days. There was a significant difference in dry mass loss among the four sampling periods (ANOVA, *F*_3,23_ = 8.311, *p* = 0.001); leaf litter breakdown gradually decreased from a peak of 68% ± 2% in July to 52% ± 4% in October (Fig. 2).

**Figure 3:**
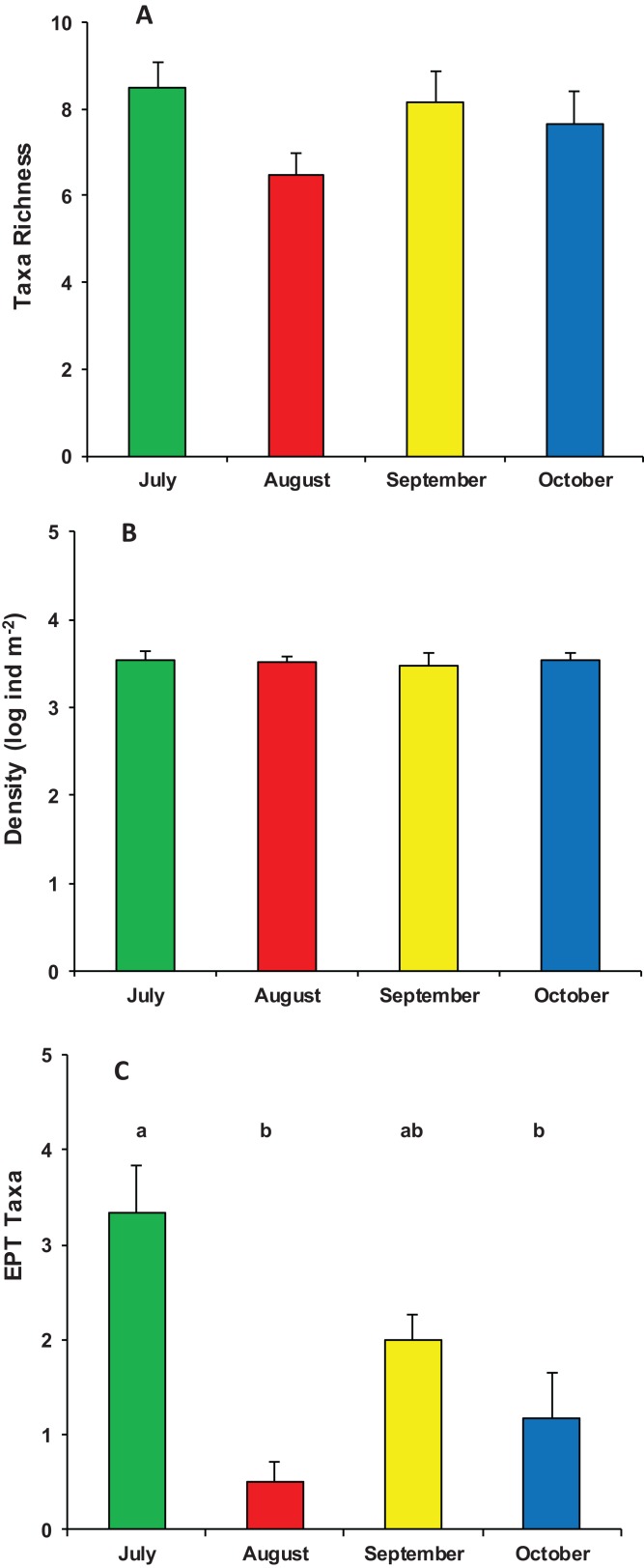
Structure of macroinvertebrate assemblages in Vera Spring. Mean (+1 SE) taxa richness (A), density (B), and number of EPT taxa (C) of macroinvertebrate assemblages in modified leaf-bags retrieved from Vera Spring after 33 days of incubation, from July 2016 to October 2016. Letters above bars indicate significant differences after Tukey’s post hoc test.

During the whole period of observation, the modified leaf-bags were colonized by 23 macroinvertebrate taxa and 5,412 individuals (Data S1). The crenobiont gastropod *Belgrandia minuscola* (Paulucci, 1881) and the amphipod *Gammarus elvirae* (Iannilli & Ruffo, 2002) dominated the assemblages with more than 80% of the individuals cumulatively collected. On average, taxa richness was 7.7 ± 1.7 taxa per bag, with a mean density of 3,758 ± 2,002 ind. m^−2^. Assemblage richness did not show significant differences among the four sampling periods (ANOVA, *F*_3,23_ = 1.870, *p* = 0.167), but it tended to be higher in July (8.5 ± 1.4 taxa) and lower in August (6.6 ± 1.2 taxa). The density of macroinvertebrate assemblages also did not vary significantly among the four sampling periods (ANOVA, *F*_3,23_ = 0.053, *p* = 0.983), although it tended to be higher in July (4,053 ± 2,699 ind. m^−2^) and lower (about 3,000 ind. m^−2^) in the three successive months. Conversely, the number of EPT taxa showed significant temporal variation (ANOVA, *F*_3,23_ = 10.126, *p* < 0.0002), with a high peak in July (3.3 ± 1.2 EPT taxa) and quite lower values in August, September, and October (Fig. 3).

**Figure 4:**
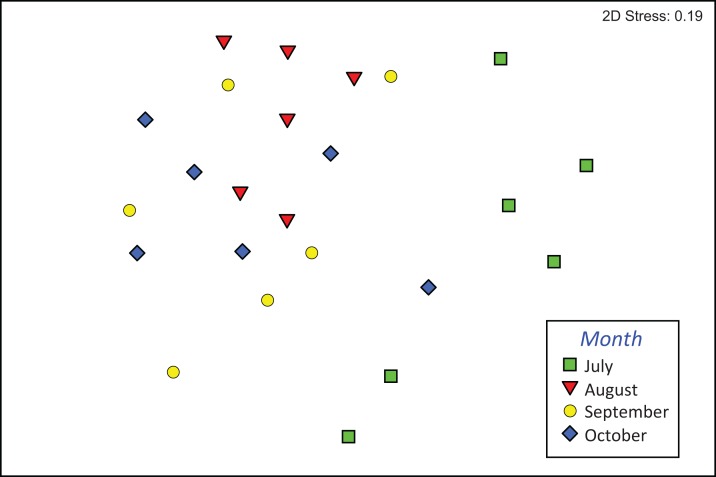
Composition of macroinvertebrate assemblages in Vera Spring. nMDS ordination (first and second axis) of assemblages composition (Bray–Curtis similarity on log (*x*+1) transformed densities) in modified leaf-bags retrieved from Vera Spring after 33 days of incubation, from July 2016 to October 2016.

The composition of assemblages varied significantly during the period of observation (PERMANOVA, pseudo-*F*_3,23_ = 3.636, *p* (perm) = 0.001). Results of pair-wise tests demonstrated that assemblages colonizing leaf material in July were significantly different from those of the three successive months (Table S1; Fig. 4).

**Figure 5:**
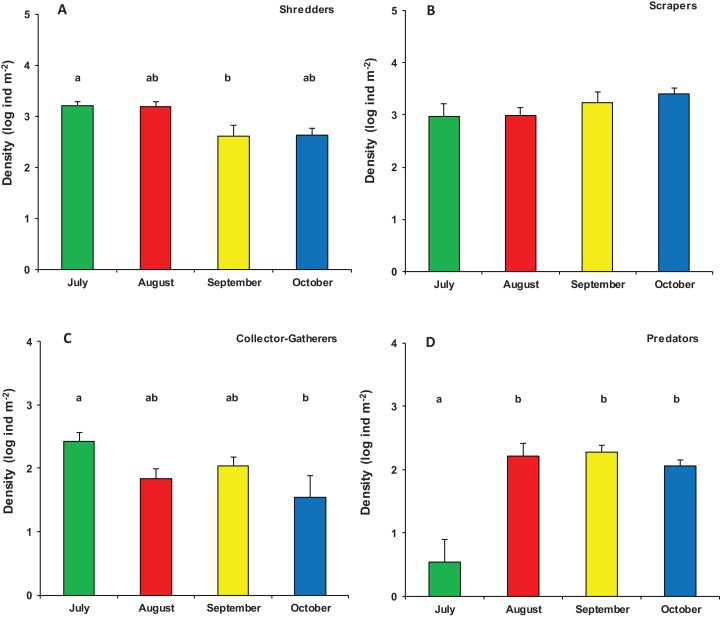
Functional organization of macroinvertebrate assemblages in Vera Spring. Mean density (+1 SE) of Functional Feeding Groups ((A) Shredders; (B) Scrapers; (C) Collector-Gatherers; (D) Predators) in modified leaf-bags retrieved from Vera Spring after 33 days of incubation, from July 2016 to October 2016. Letters above bars indicate significant differences after Tukey’s post hoc test.

Significant differences were also found in the overall functional organization of assemblages (PERMANOVA, pseudo-*F*_3,23_ = 6.982, *p* (perm) = 0.001). Compared to August, September, and October (no significant variation), the functional organization of assemblages in July was rather distinct (Table S2). This was mainly due to a higher density of shredders and, marginally, gathering-collectors. In contrast, predator’s density was significantly lower in July while temporal differences were not significant for grazers/scrapers (Table S3; Fig. 5).

## Discussion

Although we used a slightly different method and only a single measure, after 33 days of incubation the breakdown rate of poplar leaves in Vera Spring (range 0.017–0.038 day^−1^) was higher than that reported for high order streams, lakes, and floodplain ponds (range 0.0051–0.0230 day^−1^; Chergui & Pattee, 1988; Baldy, Gessner & Chauvet, 1995; Baldy et al., 2002; Bottolier-Curtet et al., 2011; Langhans et al., 2008; Pabst et al., 2008). Our *k* values were also comparable or slightly higher than those observed in third-order Mediterranean streams (range 0.010–0.037 day^−1^; Menéndez et al., 2001; Menéndez, Hernández & Comín, 2003). The faster decomposition rate of *P. nigra* leaves (0.055 day^−1^) found by Abril, Muñoz & Menéndez (2016) in an intermittent fourth-order Iberian stream was essentially due to the low incubation time (11 days) of leaf-bags (the leaves loss more mass during the first phase of the breakdown process).

Therefore, despite the constant cold temperature and the marked oligotrophic status of Vera Spring, leaf litter breakdown was faster than that of other warmer and nutrient enriched freshwater habitats. In accordance with our results, Lehosmaa et al. (2017) found that detritus breakdown of *Alnus incana* leaves in Finnish cold springs (temperature range 2.7–7.0 °C) was twofold higher than that of a mountain stream in southern Poland (Galas, 1995), while no substantial differences were found in the breakdown rate of American elm leaves between cold springs and first/second order streams in North America (Gazzera, Cummins & Salmoiraghi, 1991; Sangiorgio et al., 2010). Furthermore, Benstead & Huryn (2011) demonstrated that the breakdown rate of *Salix alaxensis* leaves in an Alaska cold spring (temperature range 3.7–7.8 °C) was similar to that found in streams at lower latitudes with temperature of about 26 °C. Therefore, we can assume that factors other than temperature and nutrients may play a key role in determining the high rate of detritus processing in cold springs. In fact, notwithstanding the stable conditions of temperature and other hydrological and physico-chemical parameters, we found significant temporal differences in the breakdown rate of poplar leaves in Vera Spring. Detritus processing was faster in July, when the density of shredder organisms was significantly higher and when the abundance of predators was distinctly lower. Therefore, according to Benstead & Huryn (2011), it seems that leaf litter breakdown is mainly driven by shredders activity. In addition, considering that predator’s diversity and abundance may significantly influence the decomposition process in freshwaters (Malmqvist, 1993; Ruetz, Newman & Vondracek, 2002; Lecerf & Richardson, 2011; Majidi et al., 2014, 2015), we can also speculate about a possible top–down control of detritus processing in Vera Spring. However, further studies in springs with different typology and extended over a larger temporal scale are needed to confirm our hypothesis.

## Conclusions

Our study adds new data on leaf litter breakdown in cold springs. Detritus processing in these ecosystems may be faster than warmer streams and other freshwater habitats. Furthermore, we found that in a thermally and chemically stable spring, seasonal variations in the rate of detritus processing could be mainly influenced by detritivore activity and biocoenotic interactions. However, the differential role of microorganism decomposers and invertebrate detritivores should be further investigated.

Although our findings are based on a single-site observation, we firmly believe that the modified leaf-bags (Di Sabatino et al., 2016, 2018) may substantially contribute to a better ecological characterization of springs, allowing a concomitant evaluation of structural and functional characteristics of these ecosystems. Further studies on detritus processing in cold springs may help to predict impacts of climate warming on freshwater ecosystem functioning and global carbon fluxes.

## Supplemental Information

10.7717/peerj.6250/supp-1 Supplemental Information 1 Raw data on abundance of Taxa and Dry mass loss of poplar leaves in Vera Spring. List and abundance of taxa and dry mass loss of poplar leaves in leaf-nets (six replicates) retrieved from Vera Spring after 33 days of incubation, from July 2016 through October 2016.

10.7717/peerj.6250/supp-2 Supplemental Information 2 Results of statistical analyses. **S1 Supplementary Material.** Results of PERMANOVA (main test and pairwise comparisons) on differences in composition of invertebrate assemblages in modified leaf-bags retrieved from Vera Spring after 33 days of incubation, from July 2016 through October 2016. Significant p-values are in bold.
**S2 Supplementary Material.** Results of PERMANOVA (main test and pairwise comparisons) on differences in densities of Functional Feeding Groups in modified leaf-bags retrieved from Vera Spring after 33 days of incubation, from July 2016 through October 2016. Significant p-values are in bold
**S3 Supplementary Material.** Results of multiple one-way ANOVA on differences in densities of Functional Feeding Groups in modified leaf-bags retrieved from Vera Spring after 33 days of incubation, from July 2016 through October 2016. Significant p-values are in bold.

## Additional Information and Declarations

### Field Study Permissions

The following information was supplied relating to field study approvals (i.e., approving body and any reference numbers): Field experiments were authorized after phone contacts by Dott. Dina Del Tosto, secretary of the managing committee of the Vera Spring natural reserve.

### Data Availability

The following information was supplied regarding data availability: Raw data on list and abundance of taxa and dry mass loss of poplar leaves in the six replicates during the four months period of observation are provided in the Supplemental File.
