# Supplementary material for: A modification of the leaf-bags method to assess spring ecosystem functioning: benthic invertebrates and leaf-litter breakdown in Vera Spring (Central Italy)
Source: PeerJ. 2019 Feb 13;7:e6250. doi: 10.7717/peerj.6250 (PMC6377591; doi:10.7717/peerj.6250)
Supplement: Supplemental Information 2 — S1 Supplementary Material. Results of PERMANOVA (main test and pairwise comparisons) on differences in composition of invertebrate assemblages in modified leaf-bags retrieved from Vera Spring after 33 days of incubation, from July 2016 through October 2016. Significant p-values are in bold. S2 Supplementary Material. Results of PERMANOVA (main test and pairwise comparisons) on differences in densities of Functional Feeding Groups in modified leaf-bags retrieved from Vera Spring after 33 days of incubation, from July 2016 through October 2016. Significant p-values are in bold S3 Supplementary Material. Results of multiple one-way ANOVA on differences in densities of Functional Feeding Groups in modified leaf-bags retrieved from Vera Spring after 33 days of incubation, from July 2016 through October 2016. Significant p-values are in bold. [file peerj-07-6250-s002.docx]

**A modification of the leaf-bags method to assess spring ecosystem functioning: benthic invertebrates and leaf-litter breakdown in Vera Spring (Central Italy).**

**Authors**

Giovanni Cristiano^a^, Bruno Cicolani^b^, Francesco Paolo Miccoli^b^, Antonio Di Sabatino^a^

**Corresponding author**

Antonio Di Sabatino Department of Life, Health and Environmental Sciences. University of L’Aquila, Via Vetoio, Coppito1, I-67100 L’Aquila, Italy.

e-mail: [antonio.disabatino@univaq.it](mailto:antonio.disabatino@univaq.it)

| **Source of variation** | **df** | **Sum of squares** | **Mean squares** | **Pseudo *F*** | ***p*(perm)** |
| --- | --- | --- | --- | --- | --- |
| Months | 3 | 5395 | 1798.6 | 3.6 | **0.001** |
| Residuals | 20 | 10002 | 500.1 |  |  |
| Total | 23 | 15397 |  |  |  |

| **Pairwise tests** | **t** | ***p*(perm)** | **perms** |
| --- | --- | --- | --- |
| July, August | 2.593 | **0.003** | 409 |
| July, September | 2.236 | **0.006** | 407 |
| July, October | 2.349 | **0.003** | 400 |
| August, September | 1.345 | 0.136 | 416 |
| August, October | 1.214 | 0.191 | 410 |
| September, October | 0.879 | 0.647 | 414 |

**Table S1 Supplementary Material**. Results of PERMANOVA (main test and pairwise comparisons) on differences in composition of invertebrate assemblages in modified leaf-bags retrieved from Vera Spring after 33 days of incubation, from July 2016 through October 2016. Significant p-values are in bold.

| **Source of variation** | **df** | **Sum of squares** | **Mean squares** | **Pseudo *F*** | ***p*(perm)** |
| --- | --- | --- | --- | --- | --- |
| Months | 3 | 1306.9 | 435.6 | 6.9 | **0.001** |
| Residuals | 20 | 1247.9 | 62.3 |  |  |
| Total | 23 | 2554.8 |  |  |  |

| **Pairwise tests** | **t** | ***p*(perm)** | **perms** |
| --- | --- | --- | --- |
| July, August | 2.886 | **0.007** | 415 |
| July, September | 3.357 | **0.008** | 407 |
| July, October | 3.560 | **0.001** | 406 |
| August, September | 1.357 | 0.165 | 413 |
| August, October | 1.720 | **0.040** | 419 |
| September, October | 1.064 | 0.383 | 409 |

**Table S2 Supplementary Material**. Results of PERMANOVA (main test and pairwise comparisons) on differences in the functional organization of invertebrate assemblages (log transformed densities of Functional Feeding Groups) in modified leaf-bags retrieved from Vera Spring after 33 days of incubation, from July 2016 through October 2016. Significant p-values are in bold

| **Source of variation** | **df** | **Sum of squares** | **Mean squares** | **F** | ***p*** |
| --- | --- | --- | --- | --- | --- |
| *Shredders* | |  |  |  |  |
| Months | 3 | 1.971 | 0.657 | 4.967 | **0.010** |
| Residuals | 20 | 2.645 | 0.132 |  |  |
| Total | 23 | 4.616 |  |  |  |
|  |  |  |  |  |  |
| *Scrapers* | |  |  |  |  |
| Months | 3 | 0.783 | 0.261 | 1.188 | 0.340 |
| Residuals | 20 | 4.392 | 0.220 |  |  |
| Total | 23 | 5.174 |  |  |  |
| *G.-Collectors* | |  |  |  |  |
| Months | 3 | 2.482 | 0.827 | 3.128 | **0.049** |
| Residuals | 20 | 5.289 | 0.264 |  |  |
| Total | 23 | 7.771 |  |  |  |
| *Predators* |  |  |  |  |  |
| Months | 3 | 12.232 | 4.077 | 14.189 | **< 0.0001** |
| Residuals | 20 | 5.748 | 0.287 |  |  |
| Total | 23 | 17.980 |  |  |  |
|  |  |  |  |  |  |

**Table S3 Supplementary Material**. Results of multiple one-way ANOVA on differences in densities of Functional Feeding Groups in modified leaf-bags retrieved from Vera Spring after 33 days of incubation, from July 2016 through October 2016. Significant p-values are in bold.
